# An Investigation of Oxides of Tantalum Produced by Pulsed Laser Ablation and Continuous Wave Laser Heating

**DOI:** 10.3390/ma17204947

**Published:** 2024-10-10

**Authors:** Alexander W. Auner, Jonathan C. Crowhurst, David G. Weisz, Zurong Dai, Kimberly B. Knight

**Affiliations:** 1 Physics Department, Boston College, Chestnut Hill, MA 02467, USA; 2 Nuclear and Chemical Science Division, Lawrence Livermore National Laboratory, Livermore, CA 94550, USA; 3 Material Science Division, Lawrence Livermore National Laboratory, Livermore, CA 94550, USA

**Keywords:** tantalum oxide, CW heating, Raman spectroscopy, TEM

## Abstract

Recent progress has seen multiple Ta_2_O_5_ polymorphs generated by different synthesis techniques. However, discrepancies arise when these polymorphs are produced in widely varying thermodynamic conditions and characterized using different techniques. This work aimed to characterize and compare Ta_2_O_5_ particles formed at high and low temperatures using nanosecond pulsed laser ablation (PLA) and continuous wave (CW) laser heating of a local area of tantalum in either air or an ^18^O_2_ atmosphere. Scanning electron microscopy (SEM) and Raman spectroscopy of the micrometer-sized particles generated by PLA were consistent with either a localized amorphous Ta_2_O_5_ phase or a similar, but not identical, crystalline β-Ta_2_O_5_ phase. The Raman spectrum of the material formed at the point of CW laser impingement was in good agreement with the previously established ceramic “H-Ta_2_O_5_” phase. TEM and electron diffraction analysis of these particles indicated the phase structure matched an oxygen-vacated superstructure of monoclinic H-Ta_2_O_5_. Further from the point of laser impingement, CW heating produced particles with a Raman spectrum that matched β-Ta_2_O_5_. We confirmed that the high-temperature ceramic phase characterized in previous work by Raman spectroscopy was the same monoclinic phase characterized in different work by TEM and could be produced by direct laser heating of metal in air.

## 1. Introduction

Tantalum pentoxide, most commonly β-Ta_2_O_5_, has been used extensively in semiconductor applications that take advantage of its wide band gap [1] and high dielectric constant [2], as well as in optical waveguides owing to its high refractive index contrast [3]. The formation of a stable ceramic polycrystalline phase of Ta_2_O_5_ has been of particular interest for use in microelectronic memory devices [4,5]. The formation of multiple phases of tantalum pentoxide has been investigated using sputtering techniques (combined with annealing up to 800 °C) [6], chemical vapor deposition (combined with annealing up to 900 °C) [7,8,9,10], annealing at less than 850 °C [4,11] or high-temperature annealing exceeding 1320 °C [4,12], and continuous wave (CW) laser heating [13,14] (along with similar CW concentrated light flux) [15] methods. The comparison of different Ta_2_O_5_ structures formed in previous studies is difficult due to the different characterization techniques employed. This work sought to provide a clear link between prominent structural characterization techniques—Raman spectroscopy, transmission electron microscopy (TEM), and electron diffraction of the multiple previously reported Ta_2_O_5_ structures formed by high- and low-temperature methods. To this end, we varied the thermal environment in which tantalum oxide was produced in air using CW laser heating and pulsed laser ablation applied to a metallic tantalum surface to form β-Ta_2_O_5_, amorphous (a-Ta_2_O_5_), and high-temperature (H-Ta_2_O_5_) phases.

A high-temperature crystalline phase of Ta_2_O_5_ was first formed in vacuum furnace experiments at temperatures around 1800 °C [12]. In that study, the transition point of the high-temperature phase was determined to be approximately 1320 °C [12]. Subsequent investigations reported conflicting high-temperature crystalline structures in large part due to the difficulty of producing H-Ta_2_O_5_ in air without using dopants [13]. However, heating with a CW CO_2_ laser, Liu et al. isolated a chemically pure crystal of H-Ta_2_O_5_ and concluded with TEM that it was tetragonal with monoclinic and orthorhombic variants [13]. An optical furnace was used by Palatinkov et al. to form a crystal that was characterized with Raman spectroscopy as ceramic Ta_2_O_5_ [15]. Light microscopy and AFM were used to investigate additional structural and mechanical properties, such as a negative coefficient of thermal expansion [16]. Raman spectroscopy was also used by Dobal et al. as a characterization technique along with XRD to identify H-Ta_2_O_5_ “crystalline” and “ceramic” samples [4]. 

The effects of annealing by slow heating and cooling rates over hours on crystalline tantalum oxide have been investigated using Raman spectroscopy [17,18], X-ray photoelectron spectroscopy [18], and X-ray diffraction [17,18]. Several low-temperature forms of crystalline tantalum pentoxide have been identified, with the β-phase being particularly well studied [17]. The formation of an a-Ta_2_O_5_ phase has been explored using electrical heating [19], chemical vapor deposition [10], ion beam sputtering [17,18,20], or pulsed laser deposition [21]. The degree to which a Ta_2_O_5_ film is amorphous or partially crystallized was found to be strongly temperature dependent [17]. Amorphous film formation was combined with post-deposition annealing with the exception of the a-Ta_2_O_5_ formed through pulsed laser deposition [21]. However, this structure has not been directly compared with β-Ta_2_O_5_ described in the aforementioned studies. Joseph et al. and Perez et al. provided a structural analysis of the β-crystalline oxide phase using Raman spectroscopy, in particular, the transformation of the amorphous phase into a crystalline structure after annealing at 700 °C [17] and above [18]. a-Ta_2_O_5_ was further elucidated by Damart et al., who used classical molecular dynamics simulations to show that it was composed of an octahedral Ta structure that can form chain-like structures, shared edges, or shared faces [22]. The vibrational modes of the measured Raman spectrum of a-Ta_2_O_5_ were theoretically calculated from the vibrational density of states [22]. Here, we show that different low-temperature crystalline and amorphous as well as modified polycrystalline high-temperature structures of Ta_2_O_5_ can be formed by nanosecond pulsed ablation and continuous wave (CW) laser heating that are unique to the physical environment of each approach. 

## 2. Materials and Methods

### 2.1. Experiment Preparation

Tantalum foil (99.9% purity, 0.25 mm thick, Sigma-Aldrich, St. Louis, MO, USA) was mounted at the laser focal point before ablation or heating by either a pulsed or a CW laser, respectively. Either laser was focused to a spot on the tantalum surface. For pulsed ablation experiments, a Q-switched Nd:YAG pulsed laser (Quantel Ultra 100, 1064 nm, shot frequency of 20 Hz, pulse width of 7 ns) ablated a single spot on the tantalum surface for 10 min (~12,000 pulses). A 10 cm biconvex lens focused the laser beam to an approximately 350 µm spot. The energy of each ablation laser pulse was estimated to be 50 mJ at the sample position (intensity of approximately 7 × 10^9^ W/cm^2^). For CW heating, a 1064 nm laser (IPG Photonics, Oxford, MS, USA) was focused by the same lens to a spot size of approximately 200 μm and estimated to be 30 W at the sample surface (intensity of approximately 1 × 10^5^ W/cm^2^). The CW laser heated the tantalum by impinging upon a single spot for one minute.

The experiments were conducted in either 1 atm of air or ^18^O_2_ (99%, Sigma-Aldrich). For experiments in an ^18^O_2_ atmosphere, the sample was mounted in a vacuum chamber where the lasers were focused through a polished sapphire window to a spot on the mounted tantalum (Figure S1). The NaCl substrate was attached perpendicular and adjacent to the mounted tantalum foil surface using a thin strip of carbon tape for pulsed ablation experiments to aid in collecting additional particles (Figure S1). After sample mounting, the chamber was immediately evacuated to <5 mTorr and backfilled with ^18^O_2_. The laser was focused onto the center of the tantalum foil to reduce the likelihood of ablating the NaCl substrate and adhesive. No traces of carbon from the adhesive were seen in subsequent Raman spectra. 

### 2.2. Ex Situ Raman Spectroscopy

The ablated tantalum surface and NaCl substrate were removed from the vacuum chamber, dismounted, and placed on an XYZ linear micrometer translation stage facing the Raman microscope objective. The 20× objective (Mitutoyo apochromatic) was used to survey the tantalum surface following laser ablation or heating with white light illumination to locate particles, as well as focus the 632.8 nm HeNe (Melles Griot LHR Laser Tube) Raman excitation laser onto identified particles. The analyzed particles had a diameter approximately equal to or greater than the focused Raman excitation laser (~10 µm spot diameter). The excitation laser power was less than approximately 10 mW at the sample surface. Backscattered light was collected through the same objective and passed through three laser rejection filters. Raman spectra were recorded with a Princeton Instruments Acton SP2300 spectrometer coupled with a Pixis 400 detector with a 300 lines/mm grating. The spectrometer was calibrated using multiple lines from a neon lamp. The estimated calibration accuracy was ±2 cm^−1^ for the 300 lines/mm grating. The acquisition times and accumulations were adjusted depending on the spectral intensity of the region of interest of each measurement and were set to a maximum of 60 s and 10 accumulations, respectively. Spectra were processed by removing background light and cosmic rays (baseline correction in the case of comparing a-Ta_2_O_5_ with β-Ta_2_O_5_) before normalizing each spectrum’s highest peak to 1 and lowest point to 0.

### 2.3. Scanning Electron Microscopy

Following Raman analysis of the ablated or heated metal surface, the tantalum target was characterized with an FEI Inspect F50 scanning electron microscope (SEM). Secondary electron images were acquired using an accelerating voltage of 5 kV for the CW ablation surface, 20 kV for the pulsed ablation surface, and an 11.5 mm working distance for both. 

### 2.4. Transmission Electron Microscopy and Electron Diffraction

TEM imaging and electron diffraction analysis were employed to identify the phase of the Ta-oxide particles. The TEM specimen was prepared using a focused ion beam (FIB) from a Ga^+^ liquid metal source in an FEI Nova 600 dual-beam electron microscope. The TEM specimen conductivity was enhanced by depositing an approximately 100 nm gold layer on the surface. A Pt strap was then applied to the sample surface using in situ deposition induced by an electron beam (e-Pt coating) and then by an ion beam (i-Pt coating) to keep the surface structure free from bombardment damage during FIB cutting. The cut section was characterized using an FEI Titan 80–300 kV S/TEM equipped with a ThermoFisher SuperX G2 EDS (energy dispersive X-ray spectroscopy) detection system for chemical analysis, operating at 300 kV. 

## 3. Results

The results of particle characterization from all experiments corresponding to the different morphologies seen from SEM imaging are summarized in Table 1.

### 3.1. SEM Imaging Overview of Tantalum Surface Morphology

**Figure 1:**
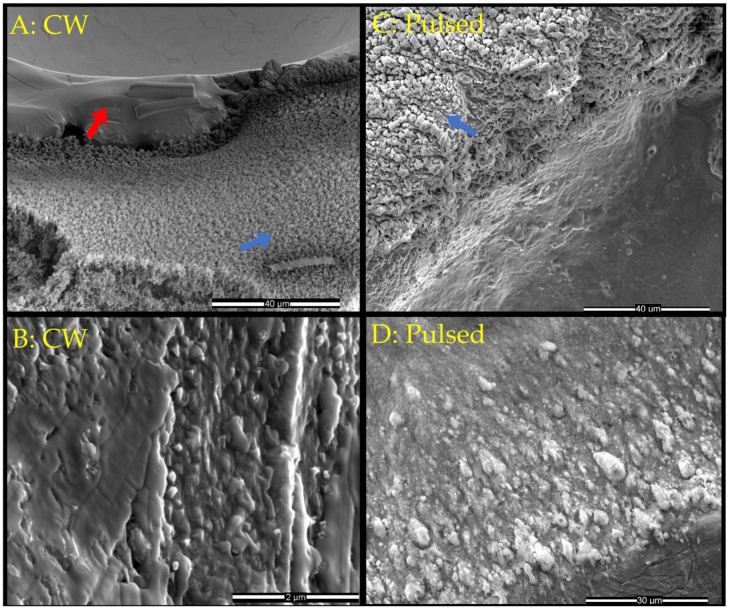
Secondary electron SEM image of tantalum surface showing laser-induced damage and oxide formation in air in (**A**) 52° tilted stage image of CW heating with red and blue arrows indicating two regions of distinct particles on and outside the crater edge, respectively. (**B**) Higher-magnification image of the crater edge and (**C**) pulsed laser ablation edge of crater. (**D**) Centimeters away from the crater center.

SEM images of the tantalum foil surface before laser impingement are provided as a reference in Figure S2. The tantalum surface was surveyed by secondary electron imaging after CW heating in air (Figure 1A,B, entire crater shown in Figure S3) or laser ablation (Figure 1C,D) to characterize the surface morphology corresponding to the different particles seen near and away from the damage area. The secondary electron image in Figure 1A shows the bottom edge of the damage area defined by a circular crater topology at the top. The crater produced by the CW laser encompassed a diameter ranging from approximately 120–150 µm. CW laser heating produced two morphologically distinct populations of particles, indicated by the red and blue arrows in Figure 1A, observed near (within microns of the smooth crater edge) and away (tens of microns from the edge) from the crater, respectively. The first population, indicated by the red arrow (found near the top of the image), appears as a smooth area outlined by a topographical interface just beyond the lip that defines the edge of the crater. To highlight this population, a higher-magnification image (Figure 1B) of the smooth section shown at the top of Figure 1A reveals irregular particles scattered throughout the smooth melt. The second population, indicated by the blue arrow, can be found highly clustered below the abovementioned topographical interface, encompassing the lower part of the image (Figure 1A). Figure 1C shows the edge of the damage crater from pulsed laser impingement. The edge of the damage crater (approximately 300 µm in diameter) formed an interface defined by fine striations adjacent to agglomerated particles. These agglomerated particles were morphologically similar to those found outside the crater produced by CW heating (Figure 1A, blue arrow). However, centimeters away from the ablation crater lay large morphologically distinct particles with a less clustered, rounded globule structure (Figure 1D). These particles were not seen in any CW-heated tantalum surface imaging.

### 3.2. Raman Identification of β-Ta_2_O_5_

**Figure 2:**
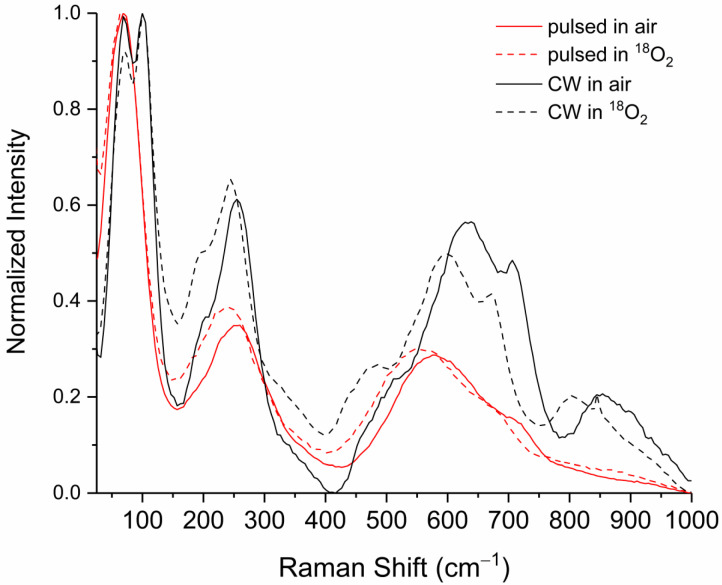
Raman spectra of crystalline particles corresponding to locations on the tantalum metal surface shown in Figure 1A,C (blue arrow) from CW and pulsed ablation, respectively. Particulates were formed in either air (solid traces) or ^18^O_2_ (dashed traces). Each spectrum’s intensity is normalized with the highest peak set to 1 and the lowest point to 0; spectra are offset along the vertical direction for clarity.

The particles seen outside the crater in Figure 1A produced by CW heating in air (blue arrow) and in the area around the crater edge in Figure 1C (blue arrow) produced by pulsed ablation (respectively) were analyzed by Raman spectroscopy (Figure 2). The Raman spectra from particles produced by CW heating were in good agreement with signatures of the Ta_2_O_5_ β-phase from the crystalline reference spectrum of Joseph et al. (assigned in Table 2) and the powder reference spectrum of Perez et al. [17,18]. While the CW particle spectrum (Figure 2, black trace) matched each of the β-Ta_2_O_5_ peaks, several weaker signatures were missing from the pulsed laser ablation particle spectrum (Figure 2, red trace)—centered at approximately 100, 200, 470, and 850 cm^−1^. 

To accurately assign Raman active modes to tantalum oxide phases, both the pulsed laser ablation and the CW heating experiments were also conducted in a pure ^18^O_2_ atmosphere to observe the spectral shift imparted by isotopic substitution. In the simplified case where we considered oscillating linear diatomic masses, the magnitude of the Raman isotopic shift could be determined from (see, for example, Weckhuysen et al. [23])

(1) $$v_{18}\approx v_{16}\sqrt{\frac{\mu_{16}}{\mu_{18}}}$$

where *ν* is the Raman shifted signature in cm^−1^ and *µ*_16_ and *µ*_18_ are the relevant reduced masses.

The isotopic shift observed in Raman spectra from particles produced by pulsed laser ablation in an ^18^O_2_ environment (dashed traces, Figure 2) above 100 cm^−1^ showed good agreement with the predicted ~5–6% shift seen for O-Ta vibrations. Below 100 cm^−1^, the isotopic shift was not perceptible. This was most likely due to the nature of the motion of the structural vibration where tantalum and oxygen atoms moved together, producing a shift that would result from the correction:

(2) $$v_{18}\approx v_{16}\sqrt{\frac{m_{Ta}+m_{16}}{m_{Ta}+m_{18}}}$$

where the shift now accounts for the combined motion of both masses in concert, *m_Ta_* is the mass of tantalum, and *m*_16_ and *m*_18_ are the relevant oxygen masses. The magnitude of this shift would then be ~0.5%, which is below the spectrometer resolution limit.

### 3.3. Raman Identification of a-Ta_2_O_5_

**Figure 3:**
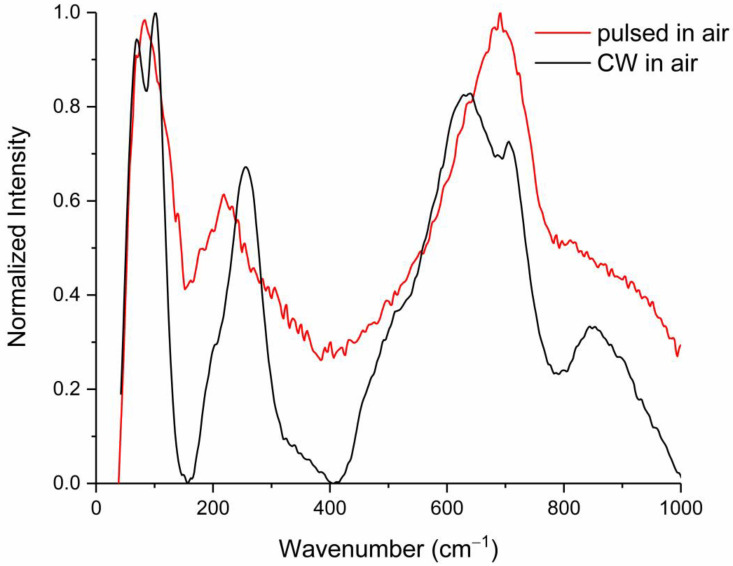
Raman spectral comparison of fine particulates from pulsed laser ablation in air on NaCl substrate (red trace) to β-Ta_2_O_5_ produced by CW heating in air on the tantalum surface (black trace). Spectra from both traces underwent polynomial baseline subtraction and were then normalized to maximum peak height.

The particles from pulsed ablation in the air found away from the crater (Figure 1D) were also present as fine, round particles on the NaCl collection substrate. Raman analysis of these particles produced broad peaks at approximately 70, 200, and 710 cm^−1^ (red trace, Figure 3 and Figure S4); these peaks were similar to the β-Ta_2_O_5_ phase produced by CW heating in air away from the crater (black trace, Figure 3). In the β-Ta_2_O_5_ phase, these overlapping peaks are assigned in Table 2 to external ionic motion, deformation, and O-3Ta stretching [4,17]. 

The broad Raman spectral peak shapes, as well as the absence of the 850 cm^−1^ stretching mode in the particles following pulsed laser ablation, were in good agreement with the Raman spectra of the amorphous phase a-Ta_2_O_5_ with the emergence of some crystal-like features seen after annealing at approximately 600–700° C in Joseph et al. [17] and Coillet et al. [20], as well as those measured and numerically simulated by Damart et al. [22]. From this comparison, these particles were identified as a disordered form in between crystalline and amorphous Ta_2_O_5_.

### 3.4. Raman Spectroscopy of Particles near the Crater of the CW-Heated Tantalum Surface

**Figure 4:**
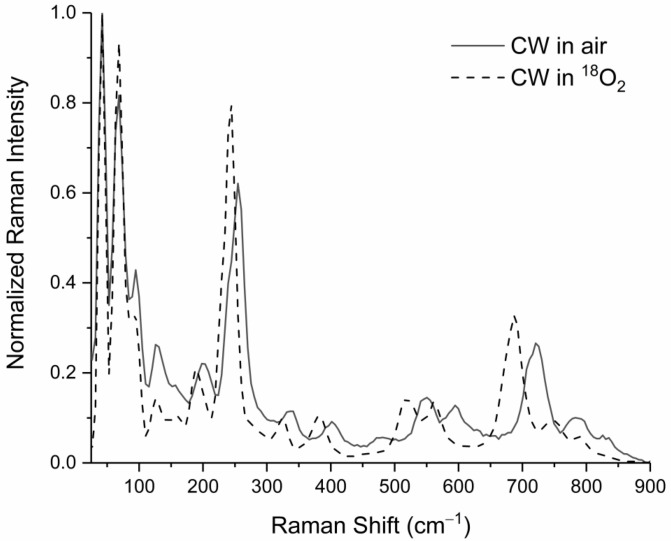
Raman spectra of particulates on the tantalum metal surface near the point of laser impingement after CW heating in either air (solid trace) or ^18^O_2_ (dashed trace).

The Raman spectrum shown in Figure 4 (solid trace) was acquired from the particle population depicted in Figure 1B (indicated by the red arrow in Figure 1A). The features in Figure 4 closely match the numerous narrow Raman signatures from a ceramic Ta_2_O_5_ phase produced in the study by Palatnikov et al. using an optical furnace [15] and by Dobal et al. by processing powders made by solid state reaction (to make ceramic samples) and laser-heated pedestal growth (to make crystals with ceramic as feed and seed material) [4]. 

The assigned motions were in good agreement with the ~6% shift seen between heating in atmospheric oxygen and heating in ^18^O_2_ in our spectra (Figure 4), for example, the Ta-O stretch that shifted from approximately 720 to 690 cm^−1^ [15]. The symmetric tetrahedron and octahedral vibrations at <126 cm^−1^ did not produce a resolvable isotopic shift.

### 3.5. TEM and Electron Diffraction Analysis of Particles near the Crater of the CW Heated Tantalum Surface

**Figure 5:**
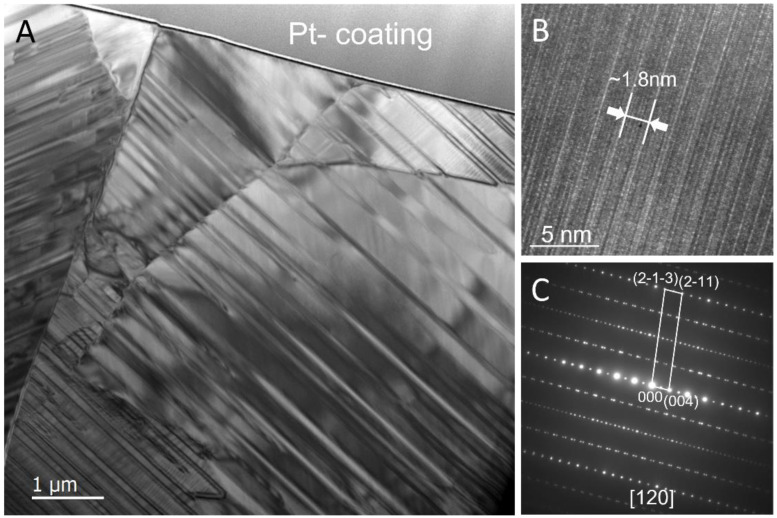
(**A**) Bright-field TEM image of a cross-section specimen prepared by focused ion beam lift out. Grain sizes across the cross-section are on the order of micrometers. (**B**) High-resolution TEM image projected along the zone axis [013] of the monoclinic Ta_2_O_5_ phase with the scale of the strong structural modulation marked. (**C**) Selected area electron diffraction pattern of the zone axis [120] with [hkl] coordinates (inset).

A thin cross-section was sliced by the focused ion beam technique from the CW crater edge shown in Figure 1A for TEM characterization (Figure 5). The cross-section was examined for morphology and to determine the material’s chemical composition and phase structure. Figure 5A shows a bright-field TEM image of the cross-section sample with gold (thin dark-contrast layer) and platinum coatings at the top. The sample displayed a morphology of polycrystalline grains with varying sizes from a few microns to tens of microns, which was similar to a casting or a sintered ceramic bulk material. We inferred from the morphology that the metal tantalum was melted at the crater area during the CW laser heating. The microstructure would then form from the solidification of the molten material. The distinct diagonal striations spaced hundreds of nanometers apart in the TEM image were due to the formation of planar crystal defects, micro-twins, and/or stacking faults.

High-resolution TEM imaging (HRTEM) and selected area electron diffraction pattern analysis were used to determine the phase structure. A series of electron diffraction patterns were taken from different grains with varying crystal orientations (Figure S5). Figure 5B,C shows an HRTEM image (Figure 5B) and the corresponding selected area electron diffraction pattern (Figure 5C), respectively. All of the electron diffraction patterns displayed the features of a single crystal (Figure 5C and Figure S5), in which all the strong base reflections in each pattern index to the monoclinic Ta_2_O_5_ phase (space group: A2/m (12), lattice parameters: a = 3.84 Å, b = 3.87 Å, c = 36.32 Å, β = 95.1°) [13,24]. The 1.8 nm lattice space marked on the HRTEM image (Figure 5B) also matched the distance of the (002) crystal plane of the monoclinic Ta_2_O_5_ phase. In addition to the strong base reflections in the diffraction pattern, weaker superlattice reflections were also observed, indicating the tantalum oxide formed here was a superstructure of the monoclinic Ta_2_O_5_ phase likely induced by oxygen vacancy ordering. EDS analysis of the same section found a stoichiometry approaching TaO_2.3_ (Figure S6 and Table S1), supporting the formation of a Ta_2_O_5_ with oxygen vacancies. EDS from TEM had the advantage over SEM because it had significantly reduced material volume dependence and nanometer-scale spatial resolution, leading to a more accurate stoichiometric determination. The stoichiometry was still an approximation when comparing the relative X-ray peak intensity due to the significant difference in atomic weight between tantalum and oxygen. TEM analysis thus confirmed that the ceramic Ta_2_O_5_ phase produced by CW heating was in good agreement with the monoclinic H-Ta_2_O_5_ from Liu et al. [13] and was the same phase identified by Dobal et al. [4] and Palatnikov et al. [16] and that was responsible for our Raman spectra shown in Figure 4.

Three types of particles were identified by Raman spectroscopy in Table 1 and summarized in Figure S4 with traces described in the legend by laser condition and distinct regions referenced relative to Figure 1. Particles produced by laser impingement in tantalum targets were as follows: β-Ta_2_O_5_ for CW heating (blue trace, Figure S4) and a disordered approximation to β-Ta_2_O_5_ laser ablation (violet trace, Figure S4), ceramic monoclinic H-Ta_2_O_5_ from CW heating (black trace, Figure S4) (also confirmed oxygen vacated with TEM), and an amorphous tantalum oxide phase from laser ablation (red trace, Figure S4).

## 4. Discussion

The morphological differences seen with secondary electron imaging between pulsed ablation and laser heating were indicative of the differences in the thermal environment. For example, the temperature of the CW-heated tantalum surface was approximately 2500 K (Figure S7). This was in contrast with material that may have formed from the vapor phase in the case of laser ablation (>10,000 K). Further, during CW heating, high temperature was sustained for tens of seconds, while the pulsed ablation plume cooled much faster. Finally, the production of H-Ta_2_O_5_ in our study and the literature appeared to rely on the thermal environment of CW heating [13,16]. In this work, the morphology of the crater and associated particles indicated a thermodynamic effect from CW heating specific to the production of different crystalline tantalum structures. In CW heating and laser ablation, similar particle morphology and structure seen outside of the crater indicated similar thermodynamic conditions outside the crater itself. The specific heating and cooling rates provided by the CW laser will be the subject of future investigation. The location of amorphous particles formed from pulsed ablation centimeters away from the crater and on the NaCl surface warrant further investigation into the correlation of particle flight time and its link to heating and/or cooling rates.

## 5. Conclusions

This work showed that the varied structures of tantalum pentoxide produced through chemical deposition or other specialized platforms can be formed through laser ablation and heating. We confirmed the different low- and high-temperature phases of Ta_2_O_5_ by Raman spectroscopy, TEM, and electron diffraction. Specifically, we confirmed that the high-temperature phase called ceramic and characterized in previous work by Raman spectroscopy [4,15] was the monoclinic phase previously characterized by TEM [13] and that can be produced by direct laser heating of metal in air. We believe the ease of providing the different laser conditions, the air atmosphere, and the ability to collect particles predictably by location will lead to further investigation into application of these phases.

## Figures and Tables

**Table 1:** Summary of particle analysis formed from pulsed laser ablation and CW heating.

Laser Type	Particulate Detected	Atmosphere	Location
Pulsed	β-Ta_2_O_5_	Air and ^18^O_2_	Crater edge
	a-Ta_2_O_5_	Air	~1 cm from the crater and on the collection substrate
CW	β-Ta_2_O_5_	Air and ^18^O_2_	~1 µm-mm from crater edge
	H-Ta_2_O_5_	Air and ^18^O_2_	Crater edge

**Table 2:** Raman peak assignments for the β-phase based on comparison with Joseph et al. [17].

Peak Assignment (cm^−1^)	Laser	Description
70	CW and pulsed	$\mathrm{External}\;\mathrm{ionic}\;\mathrm{motion},\;Ta_{x}O_{y}^{z+}$ cluster
100	CW	
200	CW	Deformation O-2Ta and O-3Ta
260	CW and pulsed	
470	CW	Stretching triple coordinated oxygen O-3Ta
610	CW and pulsed	
710	CW and pulsed	
850	CW	Stretching double-coordinated oxygen O-2Ta

## Data Availability

The data presented in this study are available on request from the corresponding author.

## Supplementary Materials

The following supporting information can be downloaded at https://www.mdpi.com/article/10.3390/ma17204947/s1: Figure S1: Laser heating and ablation setup schematic; Figure S2: SEM of surface before laser impingement; Figure S3: SEM of CW crater; Figure S4: Additional electron diffraction patterns; Figure S5: EDS tantalum lift out spectra; Figure S6: Summary of particle Raman; Figure S7: CW heating emission spectrum from tantalum surface; Table S1: Summary of EDS results.
