# Supplementary material for: An Investigation of Oxides of Tantalum Produced by Pulsed Laser Ablation and Continuous Wave Laser Heating
Source: Materials (Basel). 2024 Oct 10;17(20):4947. doi: 10.3390/ma17204947 (PMC11509264; doi:10.3390/ma17204947)
Supplement: Supplementary file 1 [file materials-17-04947-s001.zip › materials-3192866-supplementary.pdf]

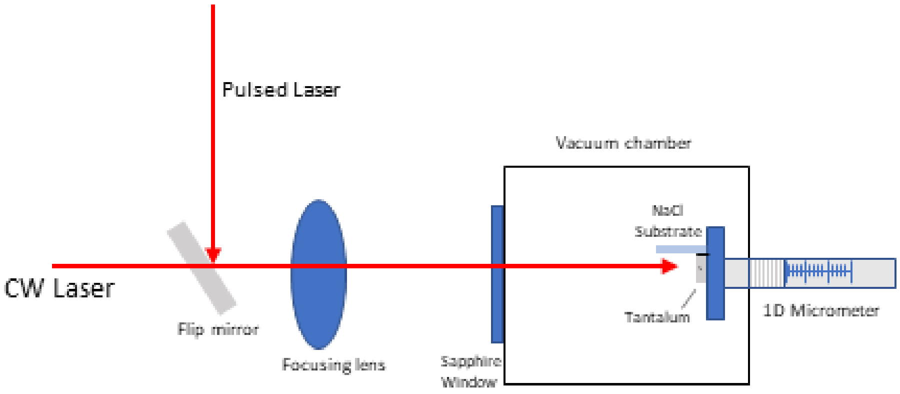

Figure S1. Schematic of the laser heating and ablation set-up used for $^{18}O_2$ experiments. Shown is a 2.75″ CF-6-way cube with one sapphire window flange, one gas inlet flange at the top (not shown), and one micrometer translation arm flange (to hold the tantalum target). NaCl substrate was attached by adhesive to the sample stage and abuts the tantalum foil. The NaCl substrate collects particulate ejected to the side.

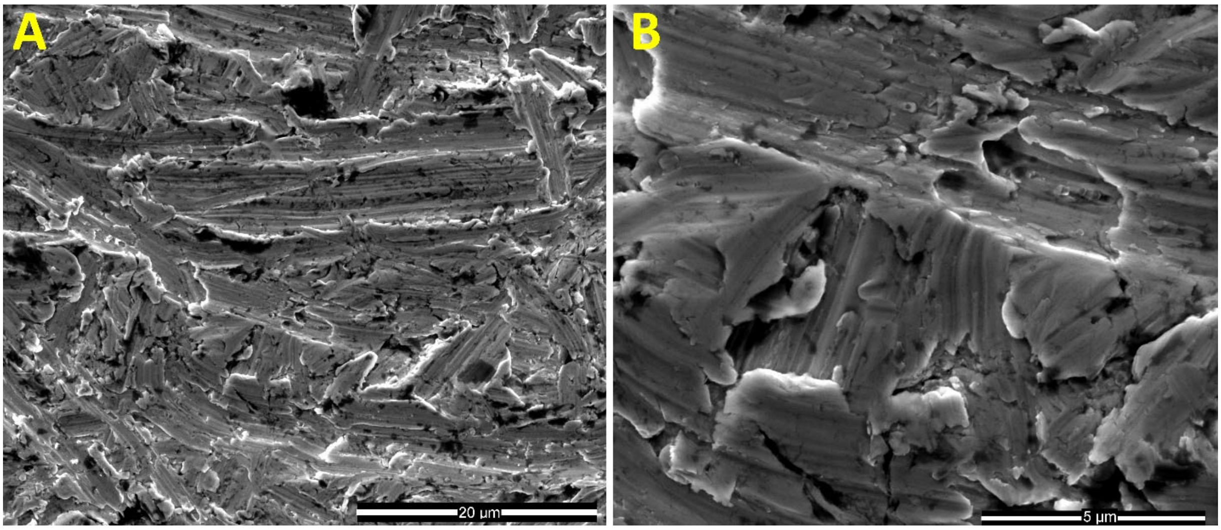

Figure S2. Secondary electron SEM image of tantalum before laser impingement at **(A)** low magnification and **(B)** high magnification.

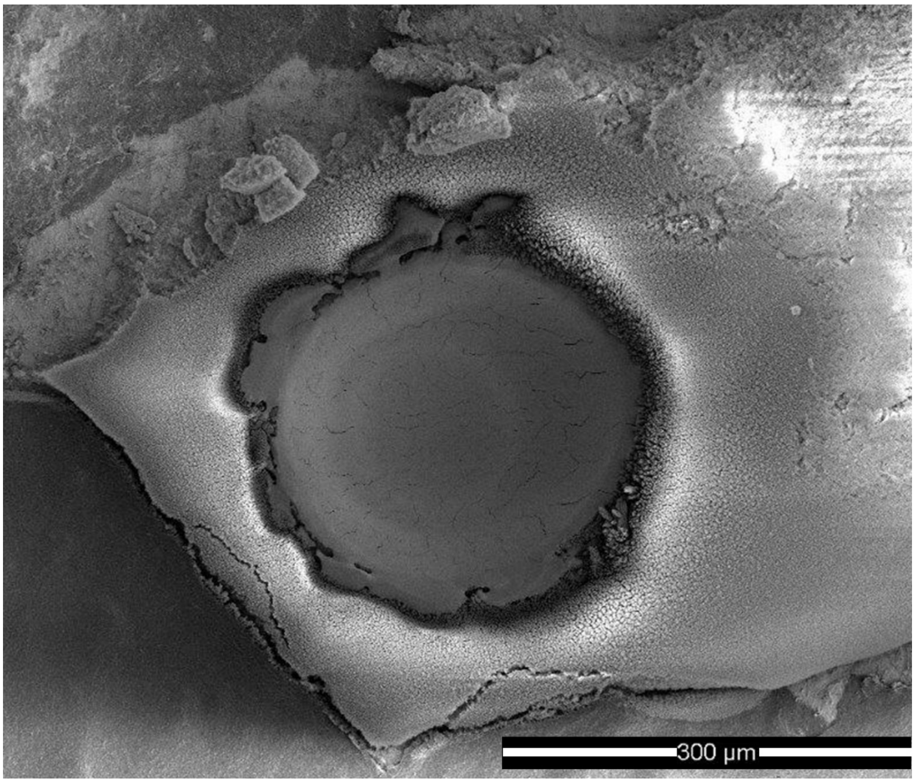

Figure S3. Low magnification secondary electron SEM image of tantalum after CW laser impingement in air showing the full crater geometry.

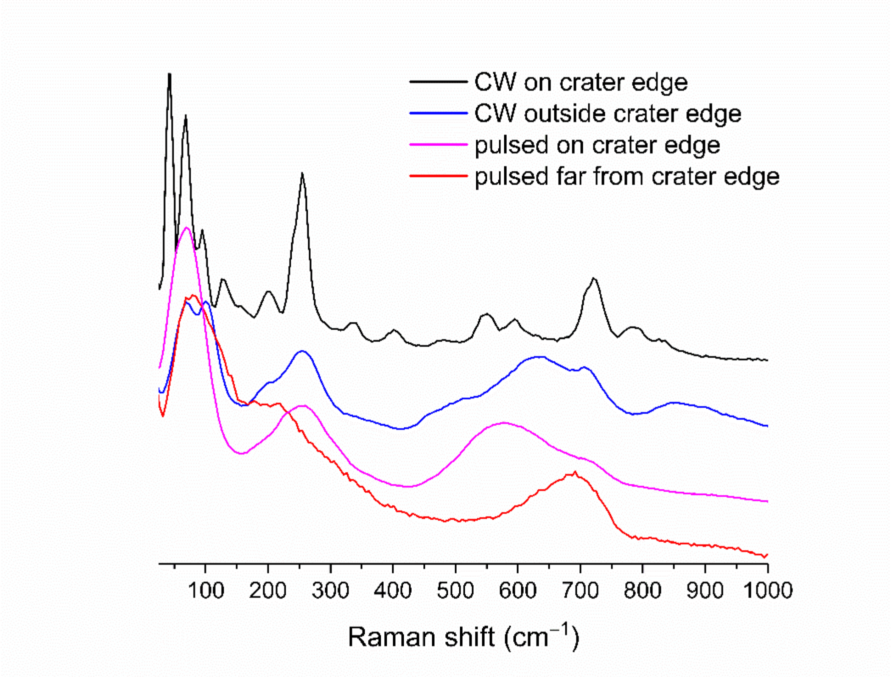

Figure S4. Raman spectral summary of each unique particulate spectrum seen formed on the tantalum surface following ablation and CW heating in air.

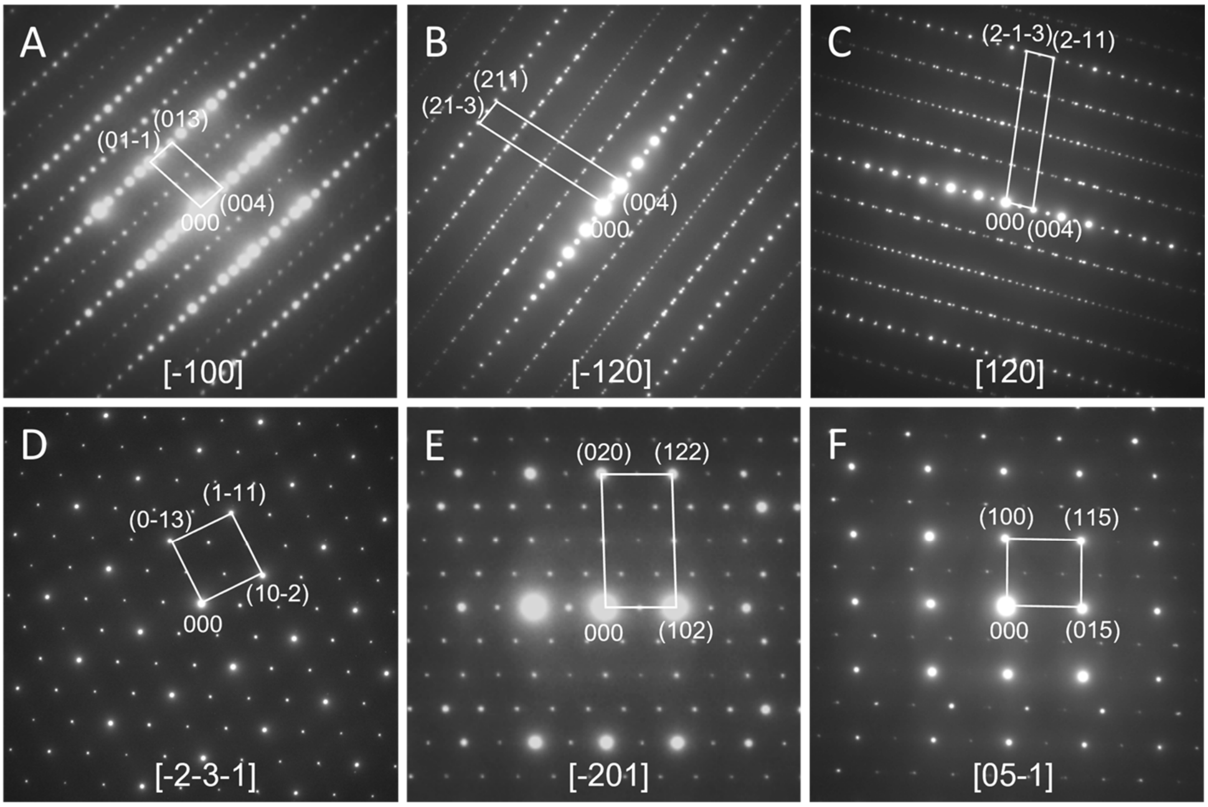

Figure S5. (**A–E**) Electron diffraction patterns taken from different grains with different crystal orientations featuring strong base reflections.

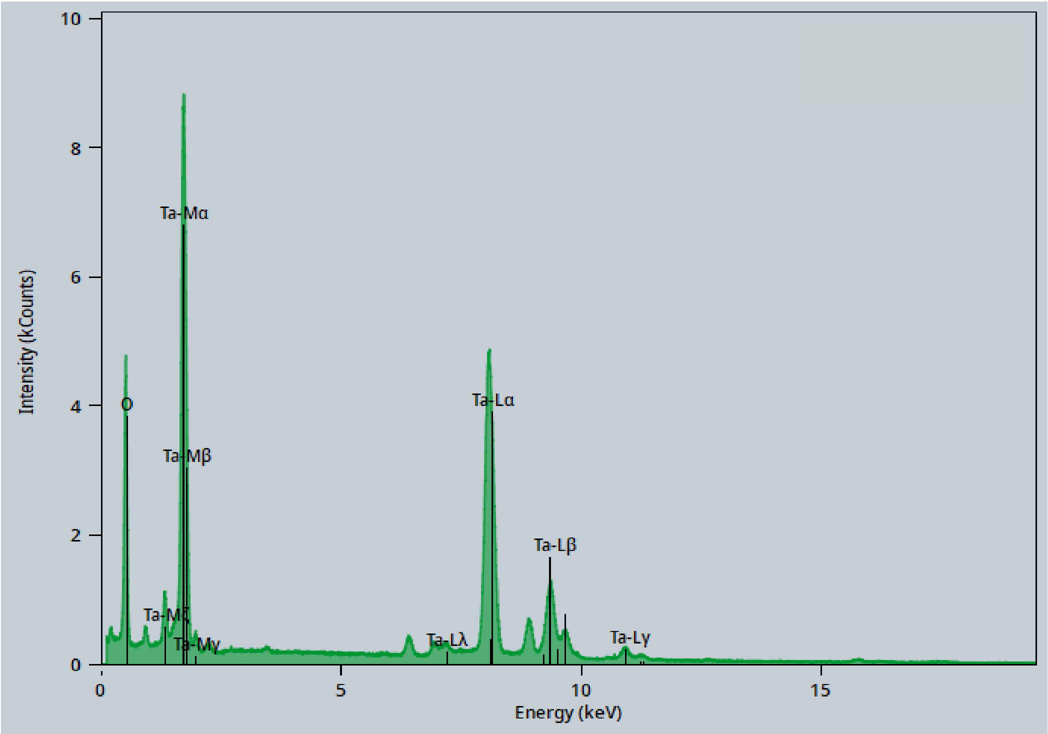

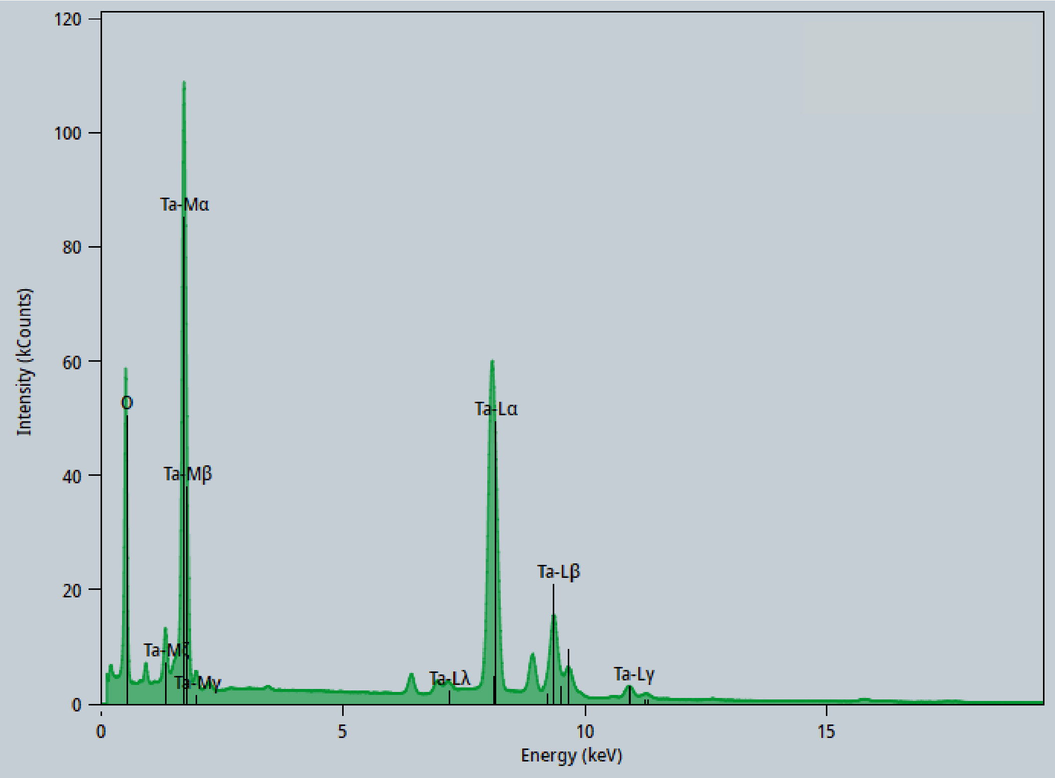

Intensity (kCounts)
120
100
80
60
40
20
0
Ta-Mα
O
Ta-Mβ
Ta-Mζ
Ta-Mγ
Ta-Lλ
Ta-Lα
Ta-Lβ
Ta-Lγ
0
5
10
15
Energy (keV)

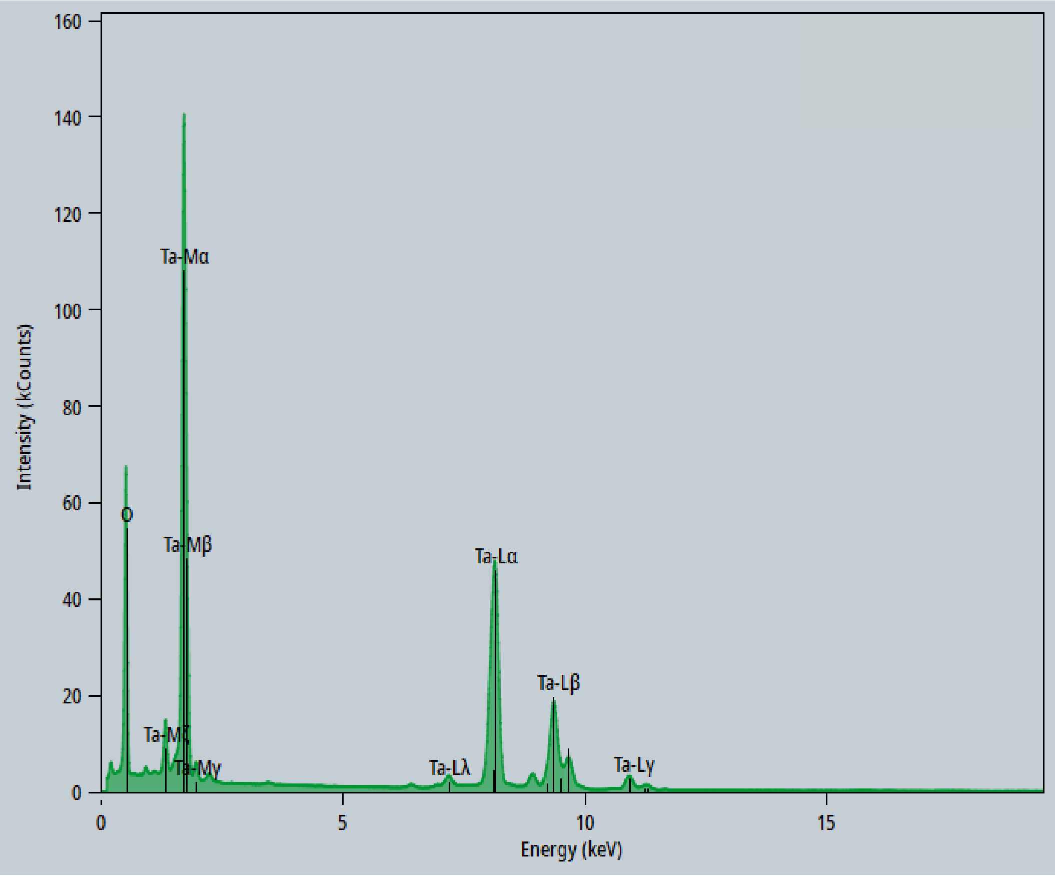

Intensity (kCounts)
160
140
120
100
80
60
40
20
0
Ta-Mα
O
Ta-Mβ
Ta-Mζ
Ta-Mγ
Ta-Lλ
Ta-Lα
Ta-Lβ
Ta-Lγ
0
5
10
15
Energy (keV)

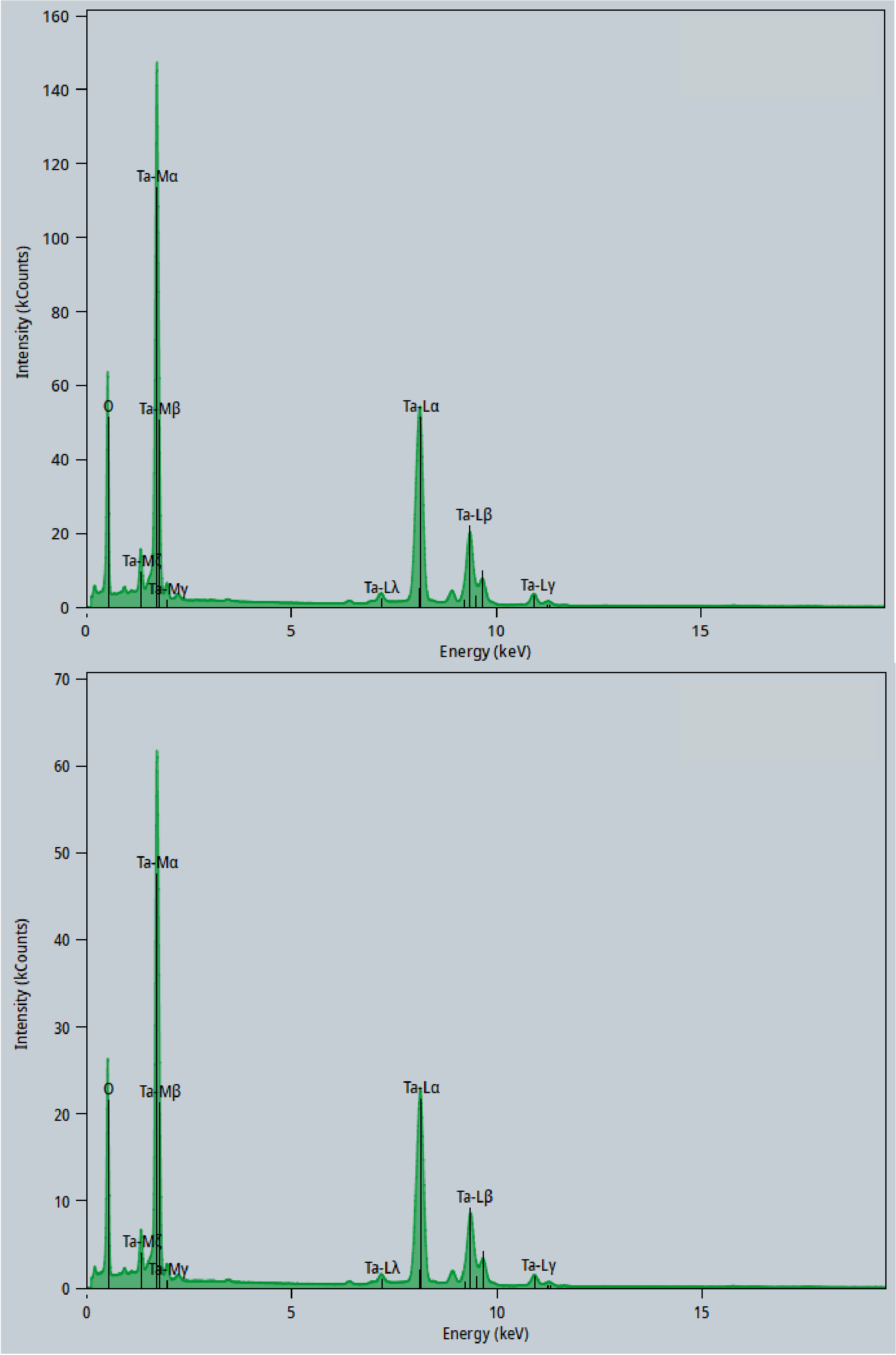

Figure S6. Energy dispersive spectrum of lift out tantalum section from four different spots.

Table S1 Summary of quantified results from the EDS spectrum shown in Figure S6 performed alongside TEM.

Element	Family	Atomic fraction (%)	Atomic error (%)	Mass fraction (%)	Mass error (%)	Fit error (%)
O	K	71	9	18	2	0.3
		71	9	18	2	0.02
		68	9	16	1	0.3
		68	9	16	1	0.7
		68	9	16	1	0.1
Ta	L	29	5	80	10	0.04
		29	5	80	10	0.02
		32	5	80	10	0.09
		32	5	80	10	0.02
		32	5	80	10	0.01

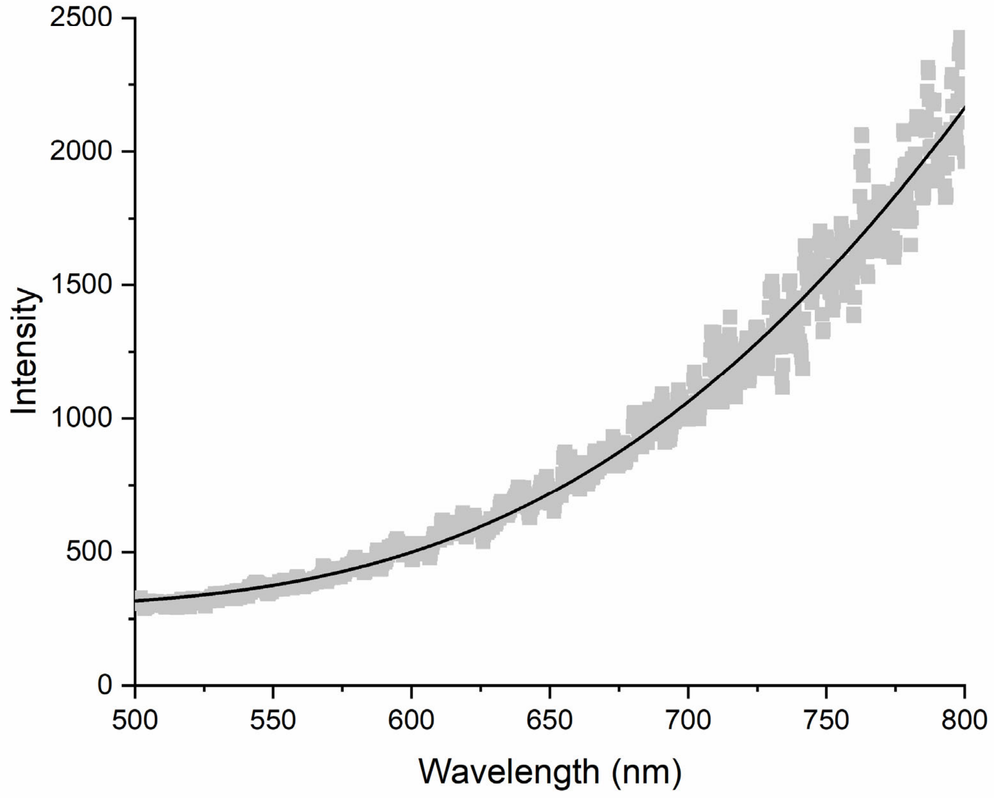

Figure S7. Blackbody emission spectrum taken from fiber probe front facing the tantalum surface. Data is fit to the Planck equation (solid black line) to determine the temperature.
